# Naso-Temporal Asymmetries: Suppression of Emotional Faces in the Temporal Visual Hemifield

**DOI:** 10.3389/fnins.2017.00014

**Published:** 2017-01-31

**Authors:** David Framorando, Mylène Bapst, Nathalie Vuille, Alan J. Pegna

**Affiliations:** ^1^Laboratory of Experimental Neuropsychology, Faculty of Psychology and Educational Sciences, University of GenevaGeneva, Switzerland; ^2^School of Psychology, The University of QueenslandBrisbane, QLD, Australia

**Keywords:** naso-temporal asymmetries, emotion, subcortical route, attentional capture, amygdala, superior colliculus, ERP, N2pc

## Abstract

An ongoing debate exists regarding the possible existence of a retino-tectal visual pathway projecting to the amygdala, which would rapidly process information involving threatening or behaviorally-relevant stimuli. It has been suggested that this route might be responsible for the involuntary capture of attention by potentially dangerous stimuli. In separate studies, anatomical evidence has suggested that the retino-tectal pathway relies essentially on projections from the nasal hemiretina (temporal visual field). In this study, we chose to take advantage of this anatomical difference to further investigate whether emotional facial expressions are indeed processed through a subcortical pathway. Using EEG, participants performed a monocular spatial attention paradigm in which lateralized, task-irrelevant distractors were presented, followed by a target. The distractors were fearful faces that appeared either in nasal or temporal visual hemifield (by virtue of their monocular presentations), while the neutral face was presented simultaneously on the opposite side. Participants were asked to identify a target letter that appeared subsequently in the nasal or temporal visual hemifield. Event-related potentials (ERPs) results revealed that fearful faces appearing in the temporal visual hemifield produced a strong inhibitory response, while a negative deflection reflecting attentional capture followed presentations of fear in the nasal hemifield. These effects can be explained by a greater sensitivity of the subcortical pathway for emotional stimuli. Fearful faces conveyed through this route are processed more effectively, consequently necessitating more vigorous suppression in order for targets to be dealt with adequately.

## Introduction

Over two decades ago, LeDoux (1996) suggested the existence of rapid subcortical pathway that conveyed information regarding threatening stimuli directly to amygdala. This route was hypothesized to bypass cortical structures, allowing these stimuli to be processed more rapidly. This phylogenetically older visual pathway may have endured in humans as it could have provided an evolutionary advantage, allowing them to respond more rapidly to stimuli that jeopardized survival. It is this pathway that is thought to be responsible for the attentional attraction of stimuli such as snakes and spiders (Öhman et al., 2001; Lipp and Waters, 2007), or emotional faces (Mogg and Bradley, 1999; Pourtois et al., 2004, 2005; Eimer and Kiss, 2007; Bannerman et al., 2009), and to be at the basis for affective blindsight (de Gelder et al., 1999; Pegna et al., 2005). A number of observations suggest that this subcortical visual pathway, running in parallel with the principal, geniculostriate route, projects information to the superior colliculus and pulvinar, and ultimately to the amygdala, which then processes emotionally significant stimuli (LeDoux, 1996; Johnson, 2005; Tamietto and de Gelder, 2010).

One interesting and potentially useful anatomical particularity of the visual system is the fact that the number of fibers connecting the retina and the superior colliculi differs depending on the hemi-retina. Indeed, lesion and autoradiographic studies in monkeys have shown that the retinotectal pathway contains an increasing number of fibers from the contralateral eye at peripheral retinal locations as one proceeds postero-medially in the colliculus, with a progression in the projections from the contralateral nasal hemiretina (Wilson and Toyne, 1970; Hubel et al., 1975). On the basis of these observations, one would expect information presented to the temporal visual hemifield (i.e., the left visual hemifield of the left eye and the right visual hemifield of the right eye) to reach the superior colliculus more readily than information presented to the nasal hemifield.

Supporting this assumption, behavioral effects of these asymmetries have been observed in studies using monocular paradigms. For example in a monocular perimetry test, babies tested between birth and 6 months responded to more peripherally located stimuli with increasing age, but differences in nasal and temporal fields were found. Indeed, the temporal field extended farther in the periphery and showed a greater sensitivity with more saccades oriented toward stimuli appearing in the temporal field than in the nasal one (Lewis and Maurer, 1992). Furthermore, this tendency remains present in adults, albeit to a lesser degree (Posner and Cohen, 1980; Lewis and Maurer, 1992). Evidence also indicates that in simple detection tasks, humans detect stimuli better when they are presented in the temporal hemifield (Osaka, 1978), suggesting that visual information is processed faster in the retinotectal than the geniculostriate visual route. Similar results were obtained with a hemianopic patient (Dodds et al., 2002) who was shown to be above chance when guessing the distance (“near” vs. “far”) of a stimulus presented in the blind *temporal* hemifield, while accuracy was at chance level for the blind *nasal* hemifield. Additionally, naso-temporal differences have been investigated in attentional capture paradigms, measured using oculomotor responses and manual reaction times in healthy controls, and have shown greater attentional capture for cues presented in the temporal, compared to the nasal hemifield (Rafal et al., 1991). Moreover, in a study with hemianopic patients, Rafal et al. (1990) found that distractors presented in the blind visual field reliably inhibited saccades toward targets in the unimpaired visual field, but only when they were presented in the blind *temporal* field and not the *nasal* one. A similar finding was later reported in 3 patients with pulvinar damage using a covert attention-shifting paradigm (Sapir et al., 2002). Here, the authors observed that orientation of attention was slower in the contralesional temporal field than the contralesional nasal field, while the reverse was found in the ipsilesional visual field.

Finally, naso-temporal differences have also been confirmed using high-density fMRI. Sylvester et al. (2007) examined the brain responses to visual stimulation in the temporal and nasal visual fields using reversing checkerboards. A significantly greater BOLD response was measured in the colliculus when stimuli were presented in the temporal field compared to the nasal field whereas no differences were found in the lateral geniculate nucleus (LGN) and in early visual cortical areas.

Interestingly, several lines of evidence have also reported naso-temporal differences using stimuli of higher biological significance, such as faces. For instance, newborns have been shown to orient their gaze preferentially to faces in the temporal compared to the nasal hemifield (Simion et al., 1998; Johnson et al., 2000). In the temporal hemifield, schematic faces were also found to be preferentially selected by 6 week-old newborns relative to non-faces, while no such difference was found in the nasal hemifield (Simion et al., 1998). Similar results were obtained with 4-month old babies (paradoxically the reverse effect was found in the nasal hemifield in this study, with inverted faces being preferred over upright faces; Johnson et al., 2000).

The orienting bias for faces presented in the temporal hemifield was also found with adults using schematic faces (Tomalski et al., 2009). In this straightforward upright/inverted schematic face detection task, saccades to upright face-like stimuli were faster relative to inverted face-like stimuli for temporal presentations, but no such difference was found for the nasal hemifield. This result can be interpreted as the consequence of an attentional capture by faces presented in the temporal hemifield.

This evidence strongly suggests that the structures involved in the rapid subcortical visual pathway (LeDoux, 1996) may be relayed mainly from the nasal hemi-retina, and may therefore rely on input essentially from the temporal visual hemifields of each eye (Rafal et al., 1990, 1991; Dodds et al., 2002; Sapir et al., 2002; Sylvester et al., 2007).

In order to test attentional deployment and its time course, one useful approach is to investigate the event-related potential (ERP) response to lateralized presentations of targets and distractors. In such procedures, a specific component has been identified, called the N2pc, which is now assumed to reflect selective spatial attention processing (Luck and Hillyard, 1994; Eimer, 1996; Woodman and Luck, 1999). The N2pc generally appears 200–300 ms after the onset of the display and is defined as an increased negative activity over occipito-parietal sites contralateral to the location of a visual stimulus. This component is observed by subtracting the values of the ipsilateral from those of the contralateral electrodes. The N2pc typically emerges during attentional selection of task-relevant stimuli (Eimer, 1996; Mazza et al., 2009). For example, in a task involving the discrimination of target letters among distractors presented above, below and to the left or right to the left of a fixation point, Eimer (1996) reported an N2pc for targets appearing laterally, suggesting that it did indeed reflect visual-spatial attention for the target. Of particular interest here, the N2pc has been also reported for fearful facial expressions revealing that the N2pc also arises during attentional attraction toward the location of biologically relevant stimuli (Eimer and Kiss, 2007).

On the basis of these findings, we reasoned that if fearful faces attract attention through a subcortical pathway, and that this pathway relies on input from the nasal hemiretina, fearful faces should therefore attract attention more efficiently when they appear in the temporal visual field. Consequently, differences in the N2pc component should be observed in spatial attention tasks when emotional faces are presented in the nasal and temporal visual field under conditions of monocular viewing.

In our task, emotional and neutral distractor faces were therefore presented in the temporal and nasal visual hemifields and were followed by a target letter on one side. The task of the participants was to discriminate the target letter that was either an “n” or an “m.” We hypothesized that fearful faces would attract attention more effectively when presented in the temporal visual hemifield due to its projections to the amygdala and would therefore produce an N2pc, which wouldn't be observed for presentations of emotional faces in the nasal hemifield.

## Methods

### Participants

Eighteen students (12 women and 6 men) from the University of Geneva took part in this study (age range: 22–28, mean = 23.93, SD = 1.8). Four subjects were removed due to excessive saccadic eye movements or eye blinks. Except one participant, all participants were right-handed as measured on the Oldfield-Edinburgh scale (Oldfield, 1971; mean laterality index: 14.6, range: 6–20) with normal or corrected-to-normal vision and had no self-declared neurological or psychological difficulties. The experiment was approved by the local ethics committee and participants gave their informed written consent prior to the procedure.

### Materials and apparatus

Eprime Professional 2.2 (Psychology Software Tools, Inc.) was used for the stimuli presentation. The stimuli were made up of 8 different identities with four male faces and four female faces. For each stimulus, two emotional expressions were used: neutral and fearful, producing a total of 16 different stimuli. Stimuli measured 6.9° by 10.2° in visual angle. Pairs of stimuli were presented on the left or right of a central fixation cross. Each pair was composed of different identities but the same gender. There were 3 conditions for the face pairs: (1) in the “nasal” condition fearful faces were presented in the nasal and neutral faces in the temporal visual field, (2) in the “temporal” condition, fearful faces appeared in the temporal visual field and neutral ones in the nasal field, finally (3) in the “control” condition, neutral faces were presented in both in the temporal and nasal visual fields. Letters “m” and “n” were used as targets and appeared either in the temporal or nasal visual hemifield.

### Procedure

Subjects were placed in a soundproof room, sitting comfortably at 50 cm from the screen. They completed the Oldfield-Edinburgh laterality questionnaire prior to the task. In order to test the nasal and temporal hemifields, we used an ocular patch placed on one eye. In this manner, by placing a patch on the left eye, the right visual half field corresponded to the temporal hemifield and the left visual half field to the nasal hemifield. Both the right and the left eyes were submitted separately to the same procedure. Half of the participants began with the right eye and the other half with the left eye.

**Figure 1:**
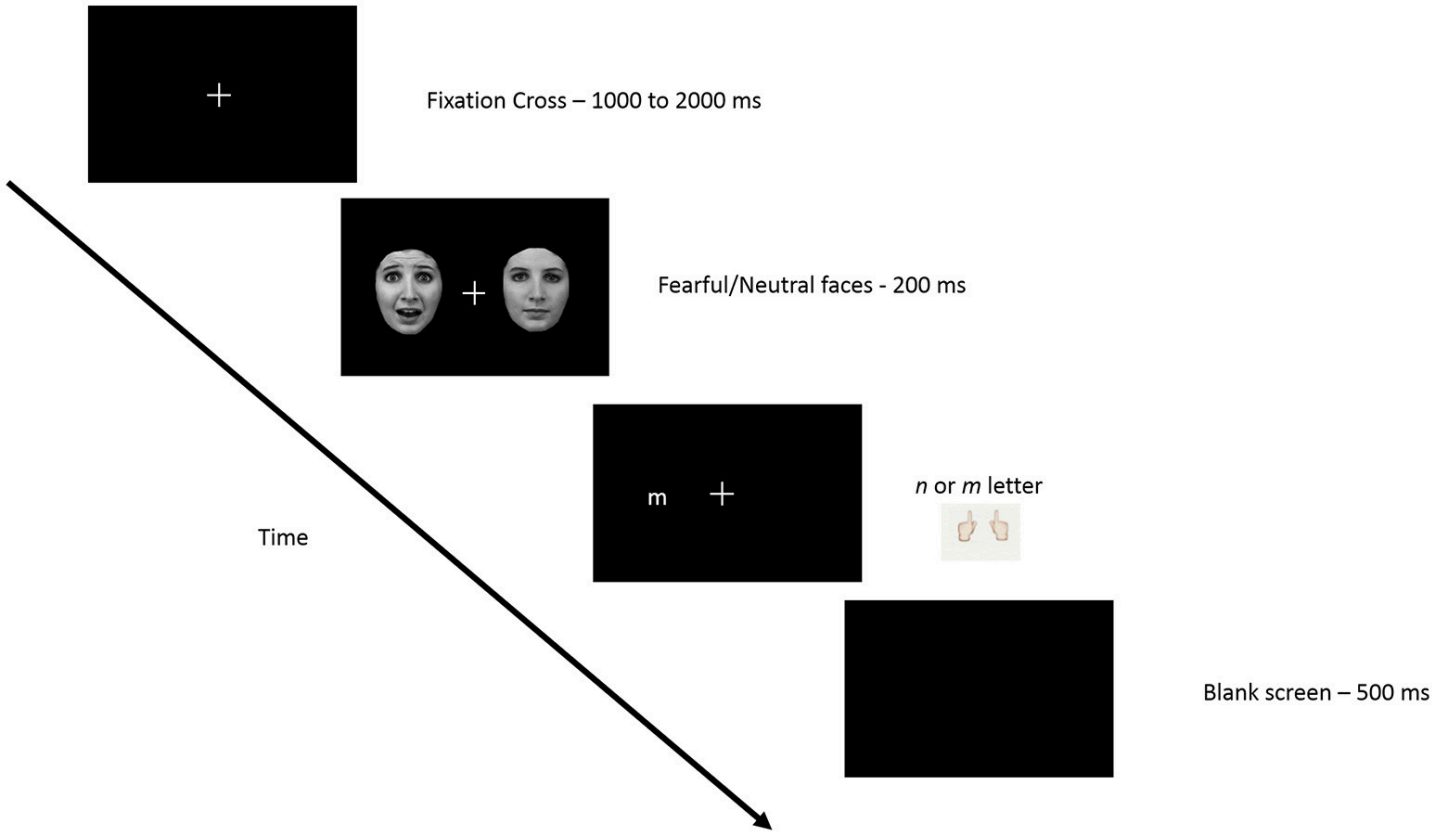
**Schematic representation of the experimental paradigm**. Procedure was carried out monocularly, such that when gaze was maintained on the fixation point, one face appeared in the temporal visual half field, and the other in the nasal visual field. After presentation of the two task-irrelevant distractor faces, a letter appeared in the temporal or nasal field to which participants were asked to respond (see text for details).

The participants' goal in this study was to determine if the letter presented on the screen was an “n” or “m” and to answer by pressing the corresponding key on a keyboard. First a fixation cross appeared in the middle of the screen for a random duration between 1000 and 2000 ms and was followed by a pair of faces that briefly (200 ms) appeared on either side of the fixation cross, centered at 9.2°. Following the disappearance of faces (0 ms), a letter (m or n), was presented at one of two positions previously occupied by the faces, also centered at 9.2° (see Figure 1). The letter remained on the screen until the participant answered. The experiment consisted of 3 conditions for face-pair presentations (temporal, nasal, and control) × 2 conditions for target presentations (temporal and nasal). Targets could therefore be validly cued (i.e., appearing at the location of a previous fearful face), invalidly cued (i.e., appearing at the opposite location of the previous fearful face) or uncued (target followed the presentation of 2 neutral faces), used here as a control condition. Thus, targets appeared in one of the 6 experimental conditions: valid temporal condition (temporal target preceded by a temporal fearful face); invalid temporal condition (temporal target preceded by a nasal fearful face); temporal control condition (temporal target preceded by 2 neutral faces face) as well as these 3 conditions for nasal target presentations. For each eye, a total of 384 trials, presented in 8 blocks of 48 trials, were delivered randomly with an equal number of trials in each of the 6 conditions. Participants were asked to maintain their gaze on the fixation cross throughout the experiment in order to avoid saccades. A 48-trial practice session was presented prior to the task in order to familiarize the subjects with the task.

### EEG recording

Continuous EEG was acquired at 1024 Hz using an AD-Box ActiveTwo amplifier (Amsterdam, The Netherlands) and 64 equally-spaced scalp electrodes referenced to the vertex. Six external electrodes EOG were placed on the face in order to monitor eye blinks and saccades (2 on the earlobes, 2 on the outer canthi of the eyes and 2 above each eyebrow).

For the EEG signal analysis, we used BrainVision Analyzer 2.1 (Brain Products, Gilching Germany). The signal was filtered between 1 and 40 Hz (Lehmann and Skrandies, 1980). Impedances were kept below 50 kΩ. Periods containing blinks, vertical eye movements (70 μV), horizontal eye movements (HEOG 50 μV) and muscular or electrical artifacts were removed from further analysis.

### Behavioral analysis

The behavioral analysis focused on reaction times and accuracy to the targets. We used the median and the mean for the analysis of respectively the reaction times and the accuracy. The temporal hemifields and the nasal hemifields of the right and the left eyes were collapsed. We ran a 3 (face location) × 2 (target location) analysis of variance (ANOVA) for repeated measures in order to determine the effect of the fearful face on the target. Additionally, *post-hoc* comparisons were performed using Tukey's HSD test.

### ERP processing

Trials with an incorrect behavioral response, as well as trials with reaction times below 200 ms and above 2000 ms were eliminated. Epochs were established from 100 ms before stimulus onset to 800 ms after stimulus onset and were baseline corrected using the 100 ms pre-stimulus period. ERPs for each of the face-pairs (nasal, temporal and control) were computed in every participant for each eye separately. In addition, ERPs for the targets were computed in each of the 6 target conditions. Grand mean ERPs were obtained by averaging the ERPs of all participants for each of these conditions in the right and left eye separately.

The peak amplitudes of the N2pc, P1, and N1 components were established by visually determining the groups of electrodes (regions of interest, or ROIs) displaying the maximum voltage and their temporal occurrence in the grand means.

**Figure 2:**
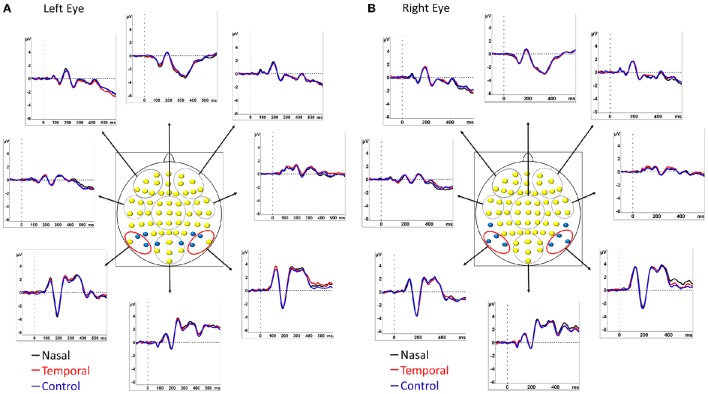
**Grand average ERP waveforms of the 14 participants**. Time zero (t0) represents the onset of the face. ERPs are shown at 8 different regions of interest (ROIs) computed by averaging the groups of electrodes contained within the red and black circles. The two ROIs used for the computation of the N2pc/Pd were composed of the 3 pairs of electrodes circled in red. Electrodes used to compute the P1 are shown in blue in **(A)** and those used to compute the N1 are shown in blue in **(B)**. ERPs are represented for left eye **(A)** and right eye **(B)** stimulation separately. Black traces correspond to presentations of fearful faces in the nasal visual hemifield and neutral faces in the temporal field; red traces correspond to presentations of fearful faces in the temporal field and neutral faces in the nasal field. The control condition in which two neutral faces were presented is indicated in blue.

Attentional attraction by the emotional face was measured on the N2pc component. The N2pc was computed using linked earlobes as the reference. The mean amplitude of two ROIs situated over the parietal leads were obtained (left ROI: PO7, P7, and P9; right ROI: PO8, P8, P10, see Figure 2 for electrode placement) in the time window of maximum activity and the amplitude contralateral to the side of appearance of the fearful face was then subtracted from the ipsilateral value. We computed this analysis separately for the left and the right presentation. We then averaged the differences of left and right presentations, creating a unique waveform for the nasal and another for the temporal conditions. As no fearful face was presented in the control condition, two control N2pcs were obtained, one “temporal” and one “nasal,” by separating the trials into odd and even segments. Odd trials were arbitrarily taken as the “temporal” conditions and even trials as “nasal” conditions. The computation was then pursued as above for the temporal and nasal conditions with fearful faces. The peak amplitudes values of the components were compared using repeated-measures ANOVAs. Additionally, *post-hoc* comparisons were performed using the of Tukey's HSD test. Violations of sphericity and *p*-values were corrected according to the epsilon of Greenhouse-Geisser or Huynh–Feldt.

For the target analysis, we measured the value of the P1 and the N1 components against the average reference on electrodes contralateral to the target only (see for example Carlson and Reinke, 2010 for a similar approach). For both these components the peak amplitudes were computed on four pairs of electrodes (for P1: PO3/PO4, PO7/PO8, P5/P6, and P7/P8; for N1 PO7/PO8, P7/P8, P9/P10, and TP7/TP8; see Figures 2A,B for electrode placement). Next, we merged the presentations of the left and the right eyes for each of the conditions, resulting in 6 ERPs per participant, one for each of our experimental conditions. The peak amplitudes values of the components were compared using repeated-measures ANOVAs. Additionally, *post-hoc* comparisons were performed using Tukey's HSD test.

## Results

### Behavioral

A repeated-measures ANOVA 3 × 2 was run in order to compare the variables related to the position of the fearful face (3: temporal, nasal and control) and the position of the letter (2: temporal and nasal). No significant differences were observed for RT (490 ms ± 48) and accuracy (92.1% ± 5) across conditions.

### Electrophysiological results

Grand mean ERPs for right and left eye presentations are shown in Figures 2A,B for the 3 face pair conditions.

### N2pc (200–230 ms)

**Figure 3:**
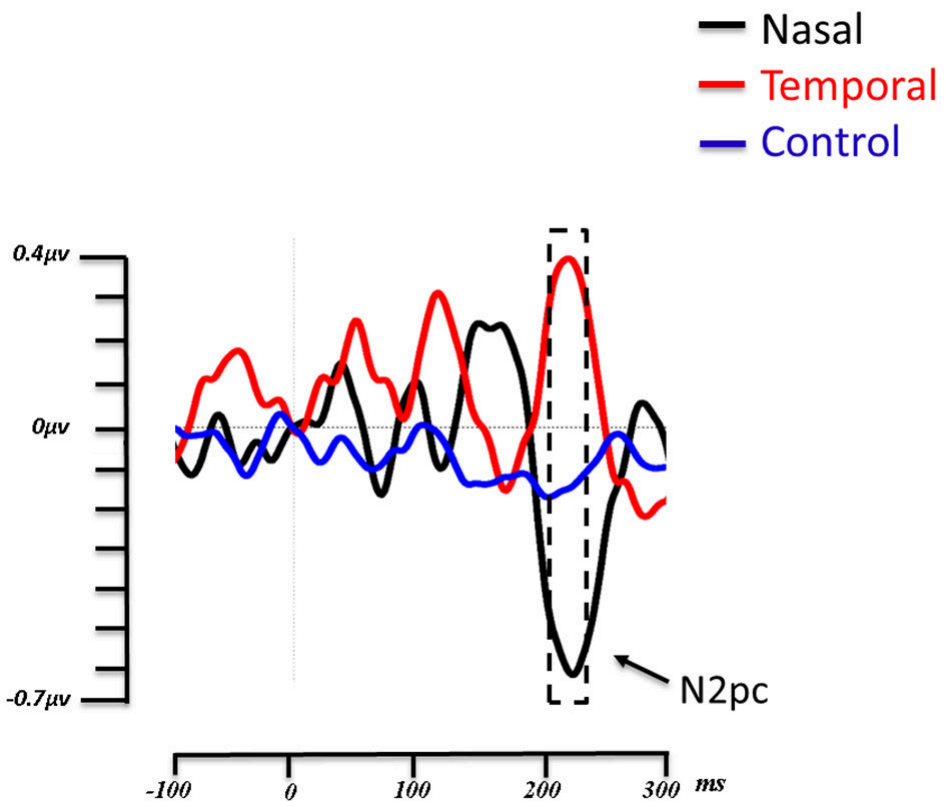
**Grand average lateralized ERP waveforms from the 14 subjects**. Time zero (t0) represents the onset of the face. The lateralized waveforms are obtained by subtracting the amplitude of the ROI contralateral to the side of presentation of the fearful faces from the ROI ipsilateral to the presentation of fearful faces. The three experimental conditions (Fear Nasal, Fear Temporal, Control) are represented, respectively in black, red and blue. For the nasal condition, a negative deflection appeared between 200 and 230 ms (indicated by the box with dashed lines). This negative deflection corresponds to the N2pc. For the temporal condition, a positive deflection appeared within the same time window, while the wave remains relatively flat in the control condition.

For the N2pc component, the time window of the amplitude analysis was 200–230 ms. Figure 3 shows the amplitude of the contralateral minus the ipsilateral ROI over time, in the control condition, as well as when fearful expressions appear in the temporal and nasal visual fields. As can be observed, the negative deflection (N2pc) is present for the nasal condition but not for the control condition. Interestingly, the temporal condition shows an opposite effect with a positive going deflection. A repeated-measures single level ANOVA was run comparing the nasal, temporal and control conditions, on the mean amplitudes in this time window, that proved to be significant [*F*_(2, 26)_ = 10.911, *p* = 0.003, η^2^*p* = 0.459]. *Post-hoc* comparisons using Tukey's HSD test revealed that the fearful faces presented in the temporal hemifield (0.34 μV ± 0.16) elicited a greater amplitude than fearful faces presented in the nasal hemifield (−0.616 μV ± −154) or in the control (−0.172 μV ± 0.048) condition (both *ps* < 0. 05; see Figure 3). The difference between the nasal and control conditions was marginally significant (*p* = 0.09).

### P1 onset of the target letter (140–170 ms)

**Figure 4:**
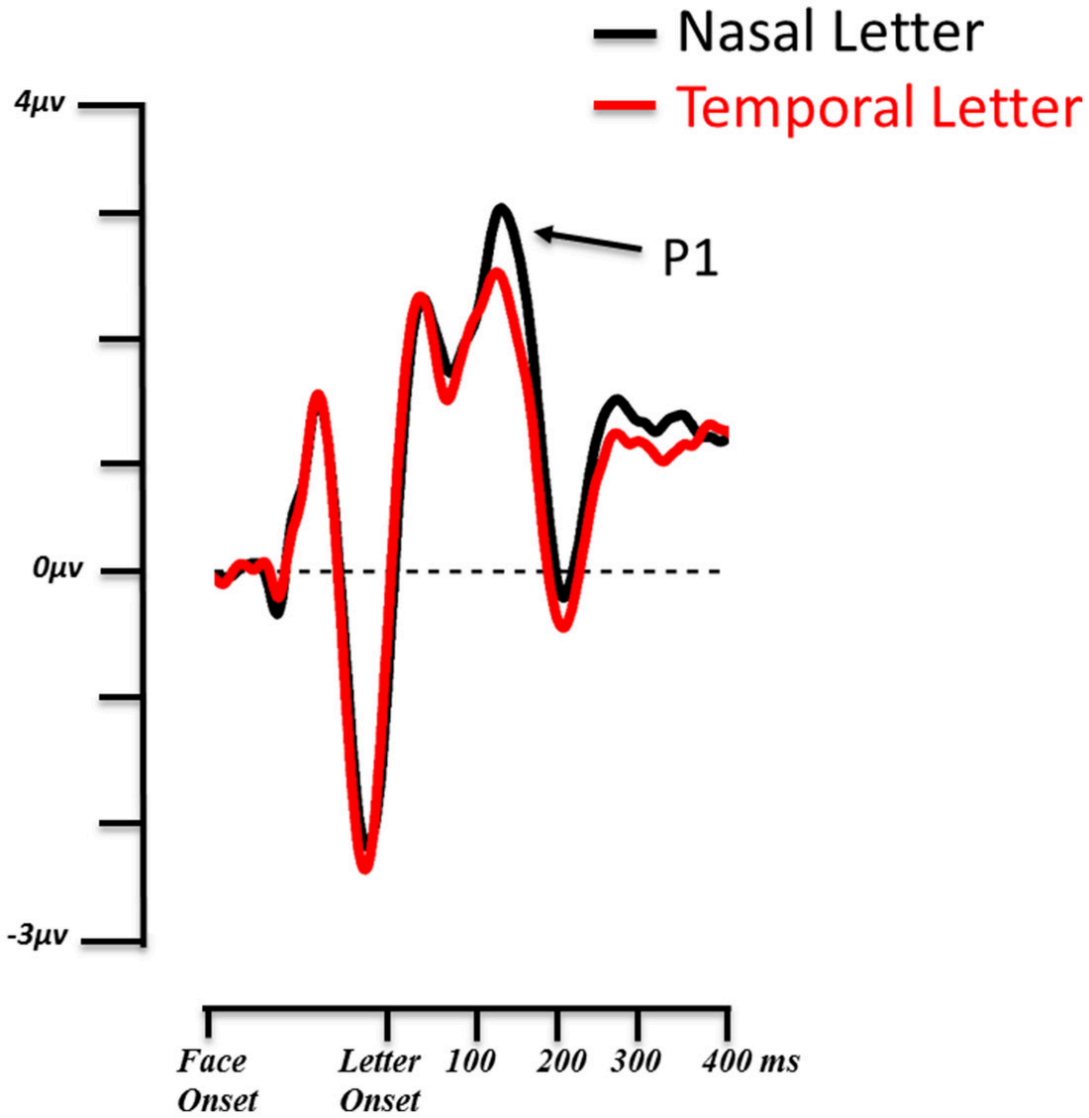
**Grand average ERP waveforms from the 14 subjects**. The P1 of the target letter is shown on the contralateral ROI for nasal (black) and temporal (red) visual field presentations. Valid and invalid conditions are collapsed.

The 3 × 2 (3: Fear Nasal, Fear Temporal, Control; 2: Letter Nasal, Letter Temporal) ANOVA revealed a main effect of target location [*F*_(1, 13)_ = 9.423, *p* = 0.009, η^2^*p* = 0.42]. The P1 was significantly larger (*p* = 0.009) when the letter appeared in the nasal hemifield (3.031 μV ± 0.506) than in the temporal hemifield (2.377 μV ± 0.474) independently of the location of the fearful face (Figure 4).

No other significant differences were observed for the P1 across conditions.

### N1 onset of the target letter (210–240)

No significant differences were observed for the N1 across conditions.

## Discussion

In the present study, we investigated the time course of cerebral processing using ERPs in an attentional capture task with emotional faces. Neutral and fearful faces were presented in the temporal and nasal visual hemifields and were followed by the target letters. We found that the N2pc was modulated by the visual hemifield of presentation of the emotional face. However, contrary to our hypothesis, the results revealed a greater N2pc when fearful faces were presented in the nasal hemifield, while a positive deflection was observed in the same time window when they were presented in the temporal hemifield. Additionally, we found that the visual ERP in response to the target letter, the contralateral P1, was increased for targets appearing in the nasal visual hemifield independently of condition.

### Lateralized event related potentials

As noted above, attentional shifting tasks using ERPs have reported that emotional faces attract attention more efficiently when competing with neutral faces (Pourtois et al., 2004, 2005; Eimer and Kiss, 2007). Eimer and Kiss (2007) presented simultaneous left and right-lateralized fearful and neutral faces, while participants attempted to detect changes in luminance at the center of the screen. They observed an N2pc contralateral to the fearful face on trials when no luminance change had occurred, suggesting that these emotional expressions captured attention, at least when no other action was required (Eimer and Kiss, 2007). The presence of an N2pc in our procedure was therefore expected. However, its manifestation for nasal field presentations of fearful faces was not. Indeed, as highlighted in the introduction, one current influential hypothesis suggests that attentional capture by emotional faces occurs via a rapid subcortical route to the amygdala (LeDoux, 1996; Johnson, 2005; Tamietto and de Gelder, 2010). Considering that this pathway relies more heavily on input from the temporal visual half field, monocular viewing should have resulted in a stronger attentional capture for fearful faces presented in the temporal field with an associated heightened N2pc. Yet the opposite effect emerged appearing to suggest that attentional capture arises for emotional faces in nasal field. An alternate explanation arises if one considers the presence of the positive deflection that arose in the same time period as the N2pc, when fearful faces were presented in the temporal field. Relatively recently, positive deflections have been described within the same time window as the N2pc, which have been shown to reflect the inhibition of attention toward a distractor. This component is known as the distractor positivity or Pd (Kerzel et al., 2011; Sawaki and Luck, 2011, 2013; Corriveau et al., 2012; Kiss et al., 2012; Burra and Kerzel, 2013, 2014; Feldmann-Wüstefeld and Schubö, 2013; Jannati et al., 2013), was evidenced by Hickey et al. (2009). In this study, the authors compared the ERP response to central targets and lateralized distractors, or the reverse (central distractors and lateralized targets). In this manner, they were able to distinguish the electrophysiological components reflecting orientation to lateral targets and suppression of lateral distractors. They observed that lateralized distractors produced a contralateral positivity in the same time period as the N2pc and hypothesized that the N2pc is actually composed of a distractors positivity associated with a target negativity. Another demonstration of the Pd was provided by Sawaki and Luck (2010) who investigated attentional deployment vs. suppression by salient singletons. In their first experiment, four letters were displayed above and below a fixation cross, extending horizontally. Before each block, one of the letters was defined as the target and either the upper or lower visual hemifield was designated as the area to attend. Participants were asked to respond to a target appearing in the attended area, however, in some trials, a salient distractor (i.e., a letter with a different color) was presented in the upper or lower visual hemifield. Results revealed an N2pc for targets presented in the attended area and a Pd for the trials in which a salient distractor appeared. The positivity observed for temporal presentations in our study thus appears to reflect a distractor positivity induced by fearful faces in this condition.

In line with this interpretation, a very recent study investigating differences in nasal and temporal field presentations for color digits reported a similar effect of distractor suppression (Huber-Huber et al., 2015). Huber-Huber et al. (2015) investigated if nasal or temporal presentations produced an attentional bias by simultaneously displaying a target color digit and a distracting digit on each side of a fixation cross. Their data yielded similar results, namely that the N2pc was greater for targets presented in the nasal visual hemifield. In a second experiment, the authors explored the role of the distractors in each visual hemifield. The distractor positivity, related to attentional suppression, was greater for temporal than nasal distractors, suggesting that temporal distractors were more actively suppressed. Since the N2pc is the subtraction of the amplitudes in the ipsilateral from the contralateral parieto-occipital electrodes on either side, an increased N2pc is the result of either an increased contralateral negativity or an increased ipsilateral positivity. In the basis of their findings, they concluded that the increased N2pc for nasal distractors did not reflect a greater attraction of attention for nasal distractors, but in fact a greater suppression for temporal distractors.

This interpretation applies equally to our study. In line with Huber-Huber et al. (2015), we argue that fearful faces are processed more efficiently when processed presented to the nasal hemiretina. These distractors are potentially more prone to interfering with target processing and thus necessitate a more active suppression though top-down inhibition. This is indexed by a distractor positivity arising over contralateral sites. On the other hand, fearful faces presented in the nasal field are likely to be processed to a lesser extent though the retino-tectal route, and therefore lead to less automatic and involuntary distraction, thus necessitating less suppression.

In our experiment the two faces that preceded the appearance of the letters were irrelevant to the task, as they provided no advance information regarding the subsequent location of the target. They were therefore only distractors that potentially interfered with attentional orientation toward the targets. Recently, Hilimire et al. (2011) examined the ERP responses to targets and salient distractors presented simultaneously. They observed that targets and distractors elicited an N2pc indicating an initial selection of the stimuli, while only salient distractors elicited a positive deflection, reflecting distractor suppression. This corroborates our explanation of a stronger distractor positivity for temporal fearful faces compared to nasal presentations.

Visual processing occurring after the presentation of the face-pairs, in particular the initial steps of target processing reflected by the P1 and N1, can also provide information regarding attentional availability (Clark and Hillyard, 1996; Pourtois et al., 2004). In particular, the P1 reflects the response to a stimulus at an attended spatial location (Luck et al., 1990; Vogel and Luck, 2000). In our experiment, we found a greater P1 for letters presented in the nasal visual hemifield independently of the position of the emotional face. This stronger P1 for nasal relative to temporal letters indirectly confirms the N2pc/Pd results. Indeed, if emotional faces presented in the temporal field produce a stronger suppression, this is likely to allow subsequent targets to be processed more efficiently. We could therefore assume that the increased P1 for nasal targets is the results of a more efficient inhibition of distractors in the temporal field. This would lead to a relatively smaller P1 for temporal stimuli than nasal stimuli due to inhibition of this location immediately after presentation of the distractors.

## Conclusion

In summary, emotional information is processed differently depending on whether it appears in the nasal or temporal visual hemifields. Fearful faces presented in temporal visual hemifield produce a contralateral positivity suggestive of a strong inhibition, while a negativity follows presentations in the nasal hemifield compatible with attentional capture. We suggest that this difference is due to the sensitivity of the subcortical pathway for emotional faces, which processes emotional stimuli more effectively in the temporal visual field, leading to a more efficient suppression of this information by the cortical structures.

## Author contributions

AP provided the idea for the study, set up the experimental procedure, and wrote the paper. DF contributed to developing the procedure, analyzed the data, and wrote the paper. MB and NV performed the recordings and participated in the analysis and in writing up the paper.

### Conflict of interest statement

The authors declare that the research was conducted in the absence of any commercial or financial relationships that could be construed as a potential conflict of interest.
